# Chain of Thought Utilization in Large Language Models and Application in Nephrology

**DOI:** 10.3390/medicina60010148

**Published:** 2024-01-13

**Authors:** Jing Miao, Charat Thongprayoon, Supawadee Suppadungsuk, Pajaree Krisanapan, Yeshwanter Radhakrishnan, Wisit Cheungpasitporn

**Affiliations:** 1 Division of Nephrology and Hypertension, Department of Medicine, Mayo Clinic, Rochester, MN 55905, USA; miao.jing@mayo.edu (J.M.); supawadee.sup@mahidol.ac.th (S.S.);; 2 Chakri Naruebodindra Medical Institute, Faculty of Medicine Ramathibodi Hospital, Mahidol University, Samut Prakan 10540, Thailand; 3 Division of Nephrology, Department of Internal Medicine, Faculty of Medicine, Thammasat University, Pathum Thani 12120, Thailand; 4 Division of Nephrology, Department of Internal Medicine, Thammasat University Hospital, Pathum Thani 12120, Thailand

**Keywords:** artificial intelligence, chain-of-thought prompting, large language models (LLMs), enhanced care, nephrology, decision-making process

## Abstract

Chain-of-thought prompting enhances the abilities of large language models (LLMs) significantly. It not only makes these models more specific and context-aware but also impacts the wider field of artificial intelligence (AI). This approach broadens the usability of AI, increases its efficiency, and aligns it more closely with human thinking and decision-making processes. As we improve this method, it is set to become a key element in the future of AI, adding more purpose, precision, and ethical consideration to these technologies. In medicine, the chain-of-thought prompting is especially beneficial. Its capacity to handle complex information, its logical and sequential reasoning, and its suitability for ethically and context-sensitive situations make it an invaluable tool for healthcare professionals. Its role in enhancing medical care and research is expected to grow as we further develop and use this technique. Chain-of-thought prompting bridges the gap between AI’s traditionally obscure decision-making process and the clear, accountable standards required in healthcare. It does this by emulating a reasoning style familiar to medical professionals, fitting well into their existing practices and ethical codes. While solving AI transparency is a complex challenge, the chain-of-thought approach is a significant step toward making AI more comprehensible and trustworthy in medicine. This review focuses on understanding the workings of LLMs, particularly how chain-of-thought prompting can be adapted for nephrology’s unique requirements. It also aims to thoroughly examine the ethical aspects, clarity, and future possibilities, offering an in-depth view of the exciting convergence of these areas.

## 1. Introduction

Large language models (LLMs), such as GPT-4, are an emergent technology that uses machine learning to process and analyze human language [1]. Initially designed to improve natural language understanding and generation, LLMs have begun to extend their applicability beyond text-based tasks like translation, summarization, or conversational agents [2]. The huge volume of data they can analyze and the quality of insight they can offer have made them valuable across a wide array of fields, from legal studies to astrophysics [2]. However, it is the field of medicine, particularly nephrology, that has seen a unique confluence of interests and technological capabilities through the deployment of these advanced LLMs [3].

LLMs are powered by deep neural networks, consisting of millions or even billions of parameters, trained on extensive datasets [2]. They have been used for sentiment analysis in marketing [4], automating document review in legal practices [5], and predicting protein folding in biochemistry [6]. With advancements in their underlying algorithms and computational power, LLMs have gained the capability to process and analyze context-rich data, paving the way for more nuanced applications. Given their multifaceted capabilities, LLMs can provide actionable insights based on patterns undetectable by human analysis alone, thereby becoming an invaluable tool in diverse disciplines [2].

Nephrology, the branch of medicine dedicated to studying kidney function, diagnosing kidney diseases, and treating conditions like chronic kidney disease, acute kidney injury, and end-stage renal disease, has its own set of intricate challenges. These range from early and accurate diagnosis to developing personalized treatment plans and managing comorbid conditions like diabetes or hypertension. The complexities often lie in the multifactorial nature of renal diseases, where genetics, lifestyle, and other health conditions play a synergistic role in influencing the course and prognosis of the disease. The integration of LLMs with nephrology is an emerging area, but initial studies and trials are showing promising results in various aspects. These include improving patient management in critical care nephrology [7], enhancing kidney transplant care [8], supporting renal diet planning [9], responding to patient inquiries [10,11,12,13], identifying pertinent nephrology literature [14,15,16], personalizing hemodialysis treatments [17], and aiding in scientific writing [18]. Utilizing the capabilities of large language models, medical professionals have the opportunity to enhance their diagnostic and treatment processes, as well as access in-depth data analyses and interpretations previously beyond reach.

Chain-of-thought prompting, a method that encourages LLMs to explain their reasoning process, acts as a catalytic function, amplifying the innate capabilities of LLMs [19,20]. By enhancing the precision and contextual comprehension of these models, it not only broadens their scope of application and increases their efficiency but also aligns them more intricately with human cognitive complexity and decision making. This approach, with its capacity to manage complex scenarios, focus on logical and sequential reasoning, and suitability for ethical and contextual judgments, becomes an invaluable asset for healthcare professionals. Its impact on improving the quality of medical care and research is anticipated to expand further.

In our review of chain-of-thought prompting in LLMs applied to nephrology, we adhered to carefully defined inclusion and exclusion criteria to ensure a focus on relevant, high-quality, and ethically sound studies. The inclusion criteria encompassed peer-reviewed articles, clinical trials, and case studies that directly engaged with chain-of-thought prompting in the context of nephrology, offering substantial empirical data or insights, and highlighting novel LLM applications in areas such as diagnostics and patient care. We limited our scope to studies published within the last five years to capture recent advancements and mandated adherence to ethical standards in AI and healthcare research. Conversely, we excluded studies that did not specifically address chain-of-thought prompting in nephrology, lacked empirical robustness, or were outdated, thereby not reflecting current practices. Additionally, unverified or non-peer-reviewed materials and studies raising ethical concerns were omitted to maintain scientific integrity and ethical compliance. This approach ensured our review was both comprehensive and aligned with the current state of research in this evolving field.

**Figure 1:**
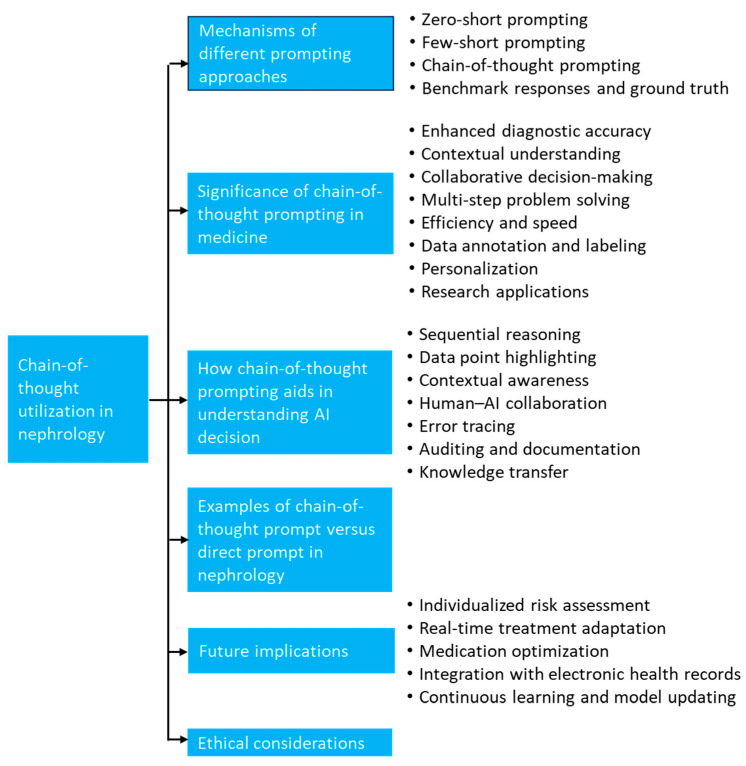
Overview of the key topics and the structural flow of the review.

This review aims to explore the application of chain-of-thought prompting in the field of nephrology (Figure 1). Notably, this review uniquely focuses on using chain-of-thought prompting in LLMs, specifically within kidney disease. It goes into detail about how this method works in nephrology and compares it with other prompt approaches. The review also looks closely at the technical side of these LLMs, like how they are built and fit into current medical systems. This provides readers with a clear understanding of LLMs’ role in healthcare. Additionally, it discusses the important ethical considerations of using LLMs in medicine, especially when dealing with sensitive patient information. The review also considers future possibilities for LLMs in treating kidney diseases. Overall, it provides new and practical insight into using advanced LLM methods in nephrology, which is not commonly covered in other reviews.

## 2. Prompting Mechanisms in Large Language Models

**Figure 2:**
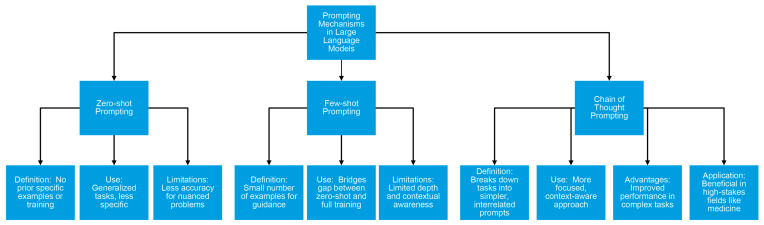
Prompting mechanisms in large language models.

LLMs like GPT-4 primarily function by comprehending human language, enabling them to handle a spectrum of tasks from basic text creation to intricate problem solving [21]. The versatility of LLMs can be significantly enhanced through the use of specialized prompting methods. Broadly, these prompting methods fall into three categories: zero-shot, few-shot, and chain-of-thought prompting (Figure 2) [22].

### 2.1. Zero-Shot Prompting

Zero-shot prompting refers to a technique used with machine learning models, especially LLMs, in which the model is asked to perform a task without any prior specific examples or training on that specific task [23]. Essentially, the model uses its pre-existing knowledge, acquired during its extensive training on a diverse dataset, to infer how to handle the new task. This ability allows the model to generate responses or solve problems in situations it has not explicitly been trained for, showcasing its generalization capabilities [24]. Recent research has validated the effectiveness of zero-shot learning across a range of standard natural language understanding tasks [25]. While zero-shot prompting is useful for generalized tasks, it often lacks the specificity and accuracy required for more nuanced or specialized problems.

### 2.2. Few-Shot Prompting

This mechanism provides the model with a small number of examples (usually just a few) to guide its understanding of a task before presenting the main query [23]. By showing the model how similar problems were solved in the past, it is prompted to solve new but related challenges. This method bridges the gap between zero-shot learning, where no examples are provided, and full-scale training.

Few-shot prompting offers more efficient in-context learning than zero-shot learning, leading to more generalized and task-specific outcomes [26]. Research indicates that performance improvement, particularly in controlling hallucinations, tends to plateau after introducing three examples in a few-shot learning scenario. Additionally, the way prompts are framed is critical; clear and straightforward prompts tend to result in higher accuracy in task execution than those that are vague or overly detailed [27]. By showing the model a few instances of the desired output, it can more effectively generalize and perform the task with limited guidance. This approach leverages the model’s pretrained knowledge and adapts it to specific tasks with minimal examples. Few-shot prompting improves task-specific performance but can still suffer from limitations in terms of depth and contextual awareness.

### 2.3. Chain-of-Thought Prompting

Chain-of-thought prompting is a strategy employed to make LLMs work in a more focused, context-aware manner [19]. Traditionally, LLMs work by predicting the next word in a sequence based on the context of the words that came before it. While they can handle quite complex queries and prompts, these models may lack the granularity to reason through intricate problems or questions, sometimes failing to maintain the semantic thread of the conversation or analysis. To overcome this limitation, chain-of-thought prompting breaks down a complex task or query into a sequence of simpler, interrelated prompts [28]. Each prompt is designed to build upon the last, thereby guiding the LLM to think “step by step” through a problem. Essentially, you are creating a “chain” of thoughts that the model follows, much like a human would when approaching a multifaceted issue. In simple terms, it is about guiding the AI to “think aloud” as it approaches a problem. This method can effectively draw out the reasoning capabilities of LLMs and shows substantial improvements in performance on math problem-solving tasks [28,29,30]. This allows for a more nuanced and contextually relevant output, which is vital for applications requiring precision and depth.

The algorithmic implementation of chain-of-thought prompting generally includes explicit prompts, training for reasoning, and sequential processing [23,31]. Initially, the model is given prompts that explicitly ask it to show its working or reasoning steps. For instance, in a math problem, instead of directly providing the answer, the model is prompted to show each step of the calculation. Training for a reasoning process might involve fine tuning the model on datasets in which the answers are accompanied by step-by-step explanations. It is notable that the model processes the problem in a sequential manner, considering one aspect of the problem at a time, much like how a human would logically break down a complex problem.

The core architecture of LLMs like ChatGPT is based on the transformer architecture [1,21]. This design is particularly adept at handling sequential data, which is essential for the step-by-step reasoning required in chain-of-thought prompting. A key feature of this architecture is its attention mechanism, allowing the model to focus on different parts of the input sequence when generating each part of the output. This is crucial for maintaining coherence in multistep reasoning. In addition, the models consist of multiple layers of transformer blocks. Each layer contributes to the model’s ability to process and generate complex sequences of text. For instance, GPT-3 has 96 layers. Finally, a high parameter count (e.g., 175 billion in GPT-3 and 1.76 trillion in GPT-4) enables the model to store and recall a vast amount of information, which is essential for the broad knowledge base required in chain-of-thought reasoning [21].

The training data for these LLMs are primarily web-based, providing a rich and varied set of texts that cover an extensive range of topics, styles, and structures [21]. This diversity helps the model to develop a broad understanding of language and context. For chain-of-thought prompting, the relevance and quality of the training data are critical. The data must include examples that demonstrate logical reasoning and step-by-step problem solving. Moreover, before training, the data undergo significant preprocessing to remove noise, correct formatting issues, and ensure they are suitable for training a high-performing model.

While a single direct prompt can sometimes yield results similar to a chain-of-thought prompt, the latter often provides more consistent, detailed, and transparent responses for complex problems [19,24,32]. It is important to note that the effectiveness of a single direct prompt versus a chain-of-thought prompt can vary depending on several factors, such as the complexity of the task; the clarity and depth of the response; the model’s training and capabilities; the predictability and consistency; and the user intent and model interpretation, as well as application-specific considerations. In certain applications, like medical decision making or technical problem solving, the step-by-step reasoning provided by chain-of-thought prompts can be particularly valuable not just for the end answer but for understanding the rationale behind it. The choice between the two approaches should be guided by the specific task at hand, the desired depth of response, and the capabilities of the LLM being used.

### 2.4. Benchmark Responses for the Comparisons of Responses across Different Prompt Approaches

When evaluating the performance of AI models such as ChatGPT in diverse learning contexts, metrics including accuracy, fluency, and coherence are commonly employed to compare the models’ responses with the established ground truth [33]. For benchmarking, researchers often use specific datasets and tasks to evaluate the performance of language models across these different approaches. The benchmark responses are usually task specific [33]. For example, a common benchmark might involve question-answering tasks, where the model’s responses are compared against a set of predefined correct answers. Ground truth refers to the accurate, real-world information or the correct answer against which the model’s output is compared. In the context of language models, ground truth is typically a set of responses or data that are known to be true or correct. These are used to evaluate the accuracy and reliability of the model’s responses. It is worth noting that these benchmarks can vary significantly based on the specific tasks, the complexity of the questions, and the domain of knowledge being tested. Additionally, the performance of a model can be influenced by its training data, architecture, and the specific techniques used for training and prompting. Each of these prompt mechanisms has its advantages and disadvantages, but for applications in high-stakes fields like medicine, chain-of-thought prompting is emerging as particularly beneficial [34].

## 3. Significance of Chain-of-Thought Prompting in Medicine

**Figure 3:**
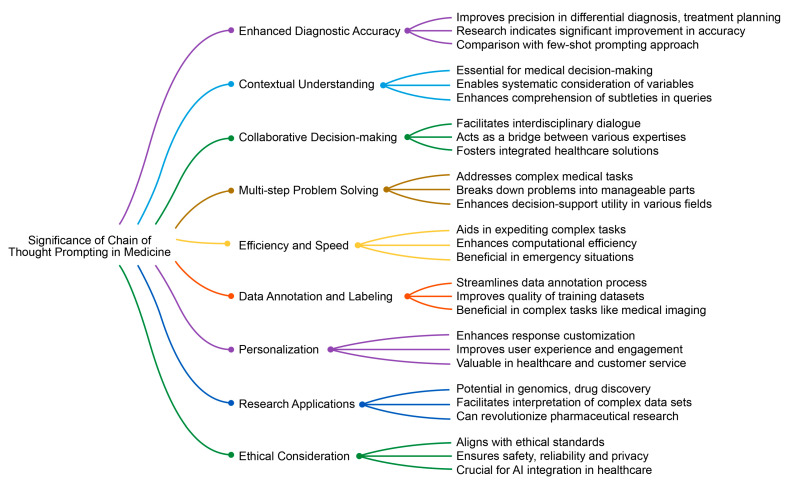
Significance of chain-of-thought prompting in medicine.

Chain-of-thought prompting can enhance the problem-solving capabilities of AI models, particularly in complex and nuanced fields like medicine. By guiding AI through a logical sequence of thoughts or steps, much like a human would think through a problem, it helps the model to process and analyze medical data more effectively. This can lead to more accurate diagnoses, better understanding of patient symptoms, and tailored treatment plans. Additionally, this approach can improve the AI’s ability to analyze and understand complex medical literature and research, thereby aiding medical professionals in keeping up to date with the latest findings and treatments. The significance of chain-of-thought prompting in medical applications extends to several key aspects (Figure 3), including those shown below.

### 3.1. Enhanced Diagnostic Accuracy

In the medical field, where the scope for error is extremely narrow and errors can have critical consequences, chain-of-thought prompting is vital for ensuring high accuracy. By guiding an LLM through a series of logical, step-by-step reasonings [32], it enhances the model’s precision, which is particularly beneficial in areas like differential diagnosis, treatment planning, and complex data interpretation. Research has shown that this method of prompting can significantly improve diagnostic accuracy [35,36], with one study indicating a 15% increase in accuracy compared to standard prompting methods [36]. However, a different study revealed that chain-of-thought prompting did not significantly outperform the few-shot prompting approach in medical question-answering tasks [26].

### 3.2. Contextual Understanding

Medical decision making relies on a deep understanding of a patient’s medical history, current conditions, and various other factors. AI strives to mimic human cognitive processes, and chain-of-thought prompting is pivotal in this context. It enables LLMs to systematically consider a range of variables, providing healthcare professionals with contextually appropriate recommendations or solutions [20,26,28]. This approach is crucial for enhancing LLMs’ ability to comprehend the subtleties of a query or problem, particularly in areas like natural language inference, machine translation, and ethical decision-making algorithms, where the context can significantly influence the outcome.

### 3.3. Collaborative Decision Making

Medical decision making frequently requires the collaboration of experts from different fields. Chain-of-thought prompting enhances this collaborative process by empowering LLMs to create sequences of thoughts or questions that steer interdisciplinary dialogue. This method transforms an LLM into a valuable instrument for facilitating complex discussions, ensuring comprehensive consideration of all pertinent aspects. It acts as a bridge between various types of expertise, aiding in synthesizing diverse perspectives and fostering a more integrated approach to healthcare solutions. By highlighting key points and prompting relevant questions, it helps maintain focus on critical issues, ensuring that all significant factors in patient care are addressed.

### 3.4. Multistep Problem Solving

Many medical tasks, such as formulating treatment plans or planning surgical procedures, require multistep reasoning. Traditional LLMs often face limitations in addressing complex problems that demand such reasoning. Chain-of-thought prompting addresses this by enabling the model to break down problems into smaller, manageable parts, process each part, and then synthesize them into a comprehensive solution [34,36]. This approach, where the problem is deconstructed into a sequence of interlinked prompts, allows the model to offer thorough solutions that encompass every phase of a medical procedure or treatment strategy. The implications of this extend beyond healthcare, finding relevance in scientific research, engineering, and complex strategy-based activities like chess, where foresight and multistep planning are key. This method not only improves the accuracy and relevance of the solutions provided but also enhances the model’s utility as a decision support tool in various fields requiring intricate, step-wise analysis.

### 3.5. Efficiency and Speed

In medical environments, where quick decision making is crucial, chain-of-thought prompting can be a valuable asset. It aids in expediting complex tasks like differential diagnosis or treatment planning, particularly benefiting less experienced clinicians or those working in settings with limited resources. Furthermore, AI algorithms can be resource intensive. By decomposing tasks into simpler components, chain-of-thought prompting enhances the computational efficiency [28]. This feature is especially beneficial in scenarios requiring rapid responses, such as in emergency medical situations or autonomous driving, where the ability to make swift, accurate decisions is paramount.

### 3.6. Data Annotation and Labeling

In the realm of supervised learning, the process of data annotation is notably laborious and time-consuming. Chain-of-thought prompting offers a solution to this challenge by directing LLMs to produce contextually precise annotations or labels [28,37]. This can substantially quicken the training of other machine learning models by providing them with accurately labeled datasets more efficiently. Expanding this approach, it can be particularly beneficial in situations where data are voluminous or complex, such as in medical imaging or natural language processing tasks, where a nuanced understanding is crucial. By automating and refining the annotation process, chain-of-thought prompting not only saves valuable time and resources but also enhances the quality of training datasets, potentially leading to more accurate and robust machine learning models.

### 3.7. Personalization

Chain-of-thought prompting enhances the ability to grasp the nuances of specific contexts, leading to more personalized responses [34,38]. This is particularly valuable in user-centric applications. Customizing responses to align with individual preferences or needs can significantly enhance the user experience and engagement. In fields such as customer service, healthcare, or education, where personalization is key, this approach enables the development of AI systems that are more responsive and attuned to the unique circumstances or requirements of each user. By delivering more targeted and relevant interactions, this not only improves satisfaction but also fosters a deeper connection and trust between the user and the AI system.

### 3.8. Research Applications

Chain-of-thought prompting holds immense potential in medical research, particularly in specialized areas like genomics, drug discovery, and epidemiological modeling. It can steer LLMs to not only generate innovative research hypotheses but also interpret complex datasets more efficiently, thus fast-tracking the advancement of medical science. This method of prompting facilitates deep, complex reasoning in LLMs, which can revolutionize areas such as pharmaceutical research [31,39]. For example, in the synthesis of new drugs, chain-of-thought prompting could guide LLMs through the intricate steps of molecule synthesis. This approach not only brings a fresh perspective to drug discovery but also significantly enhances the process, potentially leading to breakthroughs in the creation of new medications and treatments.

## 4. How Chain-of-Thought Prompting Aids in Understanding AI Decisions 

**Figure 4:**
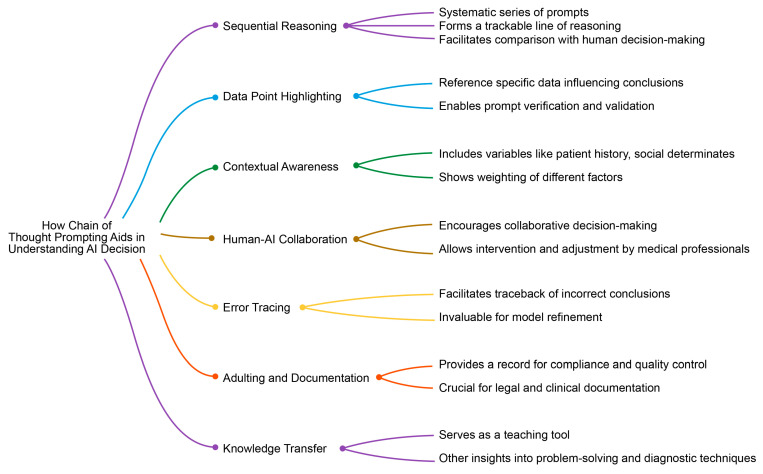
How Chain-of-thought prompting aids in understanding AI decisions.

The use of AI in critical areas such as healthcare has initiated important discussions about the need for clarity and understanding related to how these systems operate. The “black-box” nature of many AI systems, in which their decision-making process is not easily understandable, is particularly concerning. In scenarios where decisions can be a matter of life or death, it is essential to trust and understand the rationale behind an AI’s recommendations. This issue is especially relevant in healthcare, where decisions must be not only precise but also clear and defensible. This is where the concept of “chain-of-thought” prompting becomes valuable, as it offers a way to enhance decision making while also making AI systems more transparent and explainable (Figure 4).

1. Sequential Reasoning: Chain-of-thought prompting guides the model through a systematic series of prompts, where each prompt builds upon the last. This process forms a clear and trackable line of reasoning, allowing for the examination of how the model arrived at a specific conclusion. This approach is similar to the “clinical reasoning” process used by medical professionals. It facilitates the comparison between AI-generated decisions and those made by humans, making the AI’s decision-making process more relatable and understandable.
2. Data Point Highlighting: As the model progresses through its chain of thought, it frequently references specific pieces of data that influenced its conclusion. In patient care scenarios, this is akin to referencing important details from a lab report or critical symptoms from a patient’s medical history. Such referencing enables prompt verification and validation of the model’s conclusions.
3. Contextual Awareness: The model’s chain of reasoning can include contextual variables such as patient history, social determinants of health, or recent changes in medical guidelines. This shows how the model is weighing different factors, enhancing its explainability.
4. Human–AI Collaboration: Chain-of-thought prompting encourages a collaborative decision-making process. A medical professional can intervene at any point in the chain to ask for further clarification, adjust the variables, or even challenge the model’s reasoning. This two-way interaction enhances understanding and builds trust in the system.
5. Error Tracing: If the model does arrive at an incorrect or suboptimal conclusion, the chain-of-thought mechanism allows for easy traceback to identify where the reasoning went wrong. This is invaluable for both rectifying the immediate issue and refining the model for future cases.
6. Auditing and Documentation: The chain-of-thought process provides a record that can be audited for compliance and quality control. This is particularly crucial in a medical setting where documentation is paramount for both legal and clinical reasons.
7. Knowledge Transfer: For educational purposes, the chain-of-thought process can serve as a teaching tool for medical students and professionals. By observing the model’s reasoning process, they can gain insights into complex problem-solving strategies and diagnostic techniques.

## 5. Examples of Chain-of-Thought Prompts in Nephrology 

The integration of AI into healthcare has revolutionized various aspects including diagnostics, therapeutics, and patient care [3,40,41,42,43]. Nephrology, which focuses on kidney function and diseases, is particularly poised to gain significantly from this integration [44]. Given the complex range of factors influencing kidney health, nephrology requires a detailed approach for diagnosis. This complexity makes it a prime area for applying chain-of-thought prompting in AI models like GPT-4, enhancing the precision and effectiveness of diagnoses in renal diseases.

### 5.1. Regular Prompt vs. Chain-of-Thought Prompts in Diagnosing Hyponatremia due to SIADH

#### 5.1.1. Regular Prompt Methodology

In the regular prompting approach, the AI system is usually given a simple, direct prompt and provides a singular answer based on that input. For example:

- Prompt: “Patient is a 65-year-old male with a serum sodium level of 125 mEq/L and normal renal, cardiac, and liver function tests. Could this be SIADH?”
- AI’s Response: “Based on the information provided, the patient may have Syndrome of Inappropriate Antidiuretic Hormone (SIADH). Further diagnostic tests and evaluations are recommended to confirm.”

Here, the AI system’s capability is limited to answering the direct question without additional context or sequential logic. The response is a single-layered, simplified answer that does not delve into differential diagnoses, potential underlying causes, or recommended diagnostic tests and treatments.

#### 5.1.2. Chain-of-Thought Prompts Methodology

In contrast, chain-of-thought prompts involve a series of interconnected queries designed to deepen the AI’s reasoning and recommendations. Each subsequent prompt builds on the prior one, making the analysis more nuanced and context rich. For diagnosing hyponatremia due to SIADH, it would look like the following:

- First Prompt: “Patient is a 65-year-old male with a serum sodium level of 125 mEq/L and normal renal, cardiac, and liver function tests. Evaluate for SIADH.”
- AI’s First Response: “Given the low serum sodium and absence of renal, cardiac, or liver dysfunction, SIADH is a strong consideration. Next, we should look for potential triggers or causes, like medications or malignancies.”
- Second Prompt: “The patient is not taking any medications known to trigger SIADH and has no known malignancies. What should we consider next?”
- AI’s Second Response: “The absence of common triggers like medications or cancer further supports the possibility of SIADH. To confirm, additional tests such as serum and urine osmolality should be conducted. Additionally, urine sodium levels would provide further insight.”
- Third Prompt: “Urine osmolality is 450 mOsm/kg, and urine sodium is 40 mEq/L. Interpret these results.”
- AI’s Third Response: “The urine osmolality and urine sodium levels are consistent with SIADH. The next step would be to rule out other potential causes of euvolemic hyponatremia, such as hypothyroidism or adrenal insufficiency, with targeted tests.”
- Fourth Prompt: “TSH and cortisol levels are normal. What is the final assessment?”
- AI’s Fourth Response: “With normal thyroid and adrenal function tests, and given the lab values and absence of other triggers, the diagnosis of SIADH is highly likely. The next steps should involve identifying any reversible causes and initiating appropriate treatment.

Compared to the regular prompting methodology, the chain-of-thought approach provides a dynamic, multistep diagnostic pathway that emulates the complex reasoning processes required in medical diagnoses. Initially, GPT placed significant emphasis on considering a diagnosis of SIADH, advising that the next steps should focus on factors like medications or malignancies. Upon receiving information about the absence of relevant medication use and malignancy, GPT then proposed additional tests, including serum and urine osmolarity and urine sodium levels. When it was provided with data showing urine osmolality at 450 mOsm/kg and urine sodium at 40 mEq/L, GPT not only confirmed their consistency with an SIADH diagnosis but also suggested exploring potential causes of hyponatremia, such as hypothyroidism or adrenal insufficiency. Following the input of normal TSH and cortisol levels, GPT strongly supported the diagnosis of SIADH and advised identifying any reversible factors and beginning suitable treatment. Consequently, this sequential, chain-of-thought approach facilitates a more thorough evaluation of the patient’s condition, offering logical, evidence-based conclusions and guiding subsequent clinical decisions.

By leveraging chain-of-thought prompting, healthcare professionals can simulate a form of diagnostic dialogue that would otherwise occur over extended clinical discussions, thereby accelerating the diagnostic process and potentially improving patient outcomes. This method could be particularly useful in situations that require complex, nuanced reasoning such as diagnosing hyponatremia due to SIADH.

### 5.2. Regular Prompt vs. Chain-of-Thought Prompts in Diagnosing Metabolic Acidosis due to Ethylene Glycol Intoxication

#### 5.2.1. Regular Prompt Methodology

In the regular prompting approach, the AI system is usually given a simple, direct prompt and provides a singular answer based on that input. For example:

- Prompt: “35-year-old male presents with metabolic acidosis, anion gap of 25, and recent history of ingesting an unknown substance. Could this be ethylene glycol intoxication?”
- AI’s Response: “Based on the elevated anion gap metabolic acidosis and the history of unknown substance ingestion, ethylene glycol intoxication is a strong possibility. Immediate treatment and further diagnostic tests are crucial.”

#### 5.2.2. Chain-of-Thought Prompt Methodology

Chain-of-thought prompts offer a more dynamic, multistep interaction that allows for richer contextual reasoning and elaboration. It can look as follows:

- First Prompt: “35-year-old male presents with metabolic acidosis, anion gap of 25, and recent history of ingesting an unknown substance. What is your differential diagnosis?”
- AI’s First Response: “The symptoms point to an elevated anion gap metabolic acidosis, which could be caused by several factors, including ethylene glycol intoxication, methanol poisoning, or diabetic ketoacidosis among others. The ingestion of an unknown substance particularly raises concern for toxicological causes.”
- Second Prompt: “Serum glucose is 98 mg/dL, and no ketones are detected in urine. No history of diabetes. What should be the next step?”
- AI’s Second Response: “Given the absence of hyperglycemia and urinary ketones, diabetic ketoacidosis is less likely. To home in on a toxicological cause like ethylene glycol, you could perform serum osmolality and osmolal gap calculations. Additionally, sending off an ethylene glycol serum level would be diagnostic.”
- Third Prompt: “Osmolal gap is 15, and we are waiting on the ethylene glycol serum levels. What’s the interpretation?”
- AI’s Third Response: “An osmolal gap of 15 suggests the presence of an unmeasured osmotically active substance, such as ethylene glycol. While waiting for the serum levels, empiric treatment with fomepizole or ethanol should be strongly considered, given the high suspicion.”
- Fourth Prompt: “Ethylene glycol serum level comes back at 50 mg/dL. What is your assessment?”
- AI’s Fourth Response: “A serum ethylene glycol level of 50 mg/dL confirms the diagnosis of ethylene glycol intoxication. Immediate treatment with fomepizole, along with hemodialysis for toxin removal and correction of acid–base imbalances, should be initiated without delay.”

In this sequential reasoning process, GPT identified various potential causes for the patient’s elevated anion gap, particularly highlighting the history of ingesting an unidentified substance. After reviewing information that included a serum glucose level of 98 mg/dL, negative urine ketones, and no diabetes history, GPT advised calculating serum osmolality and osmolal gap and testing for ethylene glycol serum levels to explore a toxicological cause. With an osmolar gap of 15 reported and ethylene glycol serum levels still pending, GPT inferred the likelihood of an unmeasured osmotically active substance, such as ethylene glycol, being present. Although the ethylene glycol level was not yet confirmed, GPT strongly recommended starting empirical treatment with fomepizole or ethanol because of the high level of suspicion. Upon receiving a serum ethylene glycol level of 50 mg/dL, GPT verified the diagnosis of ethylene glycol poisoning and urged immediate intervention with fomepizole, complemented by hemodialysis for the elimination of the toxin and to rectify acid–base disturbances. 

The standard prompting method typically involves a straightforward, direct prompt, yielding a singular answer based on the given input. While this response is succinct, it lacks comprehensive clinical reasoning or an exploration of differential diagnoses, particularly in the case of a young man with metabolic acidosis and an anion gap of 25. In contrast, the chain-of-thought approach enables the AI to engage in a more complex, detailed discussion that mirrors the medical reasoning process. This multistep methodology aligns more closely with the complexities involved in diagnosing metabolic acidosis due to ethylene glycol intoxication, making it more useful for clinicians seeking to navigate intricate clinical scenarios.

We also conducted an evaluation of both direct prompt and chain-of-thought prompting in LaMDA and Jurassic LLMs for examples 1 and 2. On 6 February 2023, Google announced Bard, a conversational AI chatbot powered by LaMDA, a family of transformer-based neural language models [45]. Jurassic models, developed by AI21, are highly versatile, capable of both human-like text generation, as well as solving complex tasks such as question answering, text classification and many others [46]. For direct prompt, both the LamDA and Jurassic models made potential diagnosis like GPT-4. For chain-of-thought prompting, these models deliver more detailed information. However, in comparison to GPT-4, we observed that the specificity of the responses, especially from the Jurassic model, is somewhat restricted, leading to ambiguity about the subsequent steps (Supplementary Tables S1–S5).

### 5.3. Regular Prompt vs. Chain-of-Thought Prompts in Diagnosing Hypertension due to Fibromuscular Dysplasia of Renal Artery

#### 5.3.1. Regular Prompt Methodology

In the standard prompting methodology, the question posed to the AI is straightforward and aims for a concise answer. Example:

- Prompt: “A 32-year-old female presents with severe hypertension not controlled by multiple antihypertensive medications. Could this be due to fibromuscular dysplasia of the renal artery?”
- AI’s Response: “Given the young age and severe, resistant hypertension, fibromuscular dysplasia affecting the renal artery could be a consideration. Further imaging studies are recommended to confirm.”

#### 5.3.2. Chain-of-Thought Prompt 

The chain-of-thought prompts approach allows for an extended dialogue that can better capture the complexities of medical diagnosis and treatment. For example:

- First Prompt: “A 32-year-old female presents with severe hypertension resistant to multiple antihypertensive medications. What is your differential diagnosis.”
- AI’s First Response: “In a young female with resistant hypertension, the differential diagnosis should include secondary hypertension causes such as renal artery stenosis due to fibromuscular dysplasia, pheochromocytoma, primary hyperaldosteronism, or Cushing’s syndrome. Initial workup should aim to narrow down these possibilities.”
- Second Prompt: “All endocrine workup including plasma metanephrines, aldosterone-to-renin ratio, and dexamethasone suppression test are within normal limits. What should be the next diagnostic step?”
- AI’s Second Response: “Since endocrine causes have been ruled out, imaging studies focusing on the renal vasculature should be the next step. Renal artery Doppler ultrasound can be a noninvasive starting point, but CT angiography or MR angiography may provide more definitive information.”
- Third Prompt: “A beaded appearance is noted in the renal artery on a CT angiogram. What’s your assessment?”
- AI’s Third Response: “A beaded appearance of the renal artery is characteristic of fibromuscular dysplasia. This likely explains the patient’s resistant hypertension. Interventional approaches like renal artery angioplasty should be considered.”
- Fourth Prompt: “Patient undergoes successful angioplasty but still has moderately elevated BP. What is your management plan?”
- AI’s Fourth Response: “Even after successful angioplasty for fibromuscular dysplasia, some patients may continue to have elevated blood pressure. Management should focus on optimizing antihypertensive medication regimens, regular follow-up imaging to ensure no restenosis, and lifestyle modifications

The response from regular prompting is concise but lacks depth in clinical reasoning or a thorough exploration of differential diagnoses for a young person with resistant hypertension. On the other hand, the chain-of-thought approach offers a detailed, stepwise discussion that emulates the clinical reasoning process. This approach is particularly valuable as a diagnostic aid for complex conditions, such as hypertension caused by fibromuscular dysplasia of the renal artery. By engaging in this multistep interaction, the assessment gains a deeper dimension, assisting clinicians in both the diagnosis and the planning of subsequent management strategies.

## 6. Future Implications of Chain-of-Thought Prompting in Crafting Personalized Treatment Plans for Kidney Disease 

### 6.1. Individualized Risk Assessment

Using chain-of-thought prompting for kidney diseases marks a major change in risk assessment methods. Conventionally, renal conditions are often assessed using generalized algorithms that tend to overlook patient-specific variances. The utility of chain-of-thought prompting is evident when applied sequentially, ingesting clinical, laboratory, and lifestyle data in a structured manner. By doing so, it refines existing algorithms and prognostic models, enhancing specificity and sensitivity for renal pathologies, such as chronic kidney disease, acute renal failure, and hypertensive nephrosclerosis. This personalized approach allows for a dynamic recalibration of risk, potentially preventing the onset or progression of kidney disease at an early stage. 

### 6.2. Real-Time Treatment Adaptation

Chain-of-thought prompting does not merely stop at risk assessment; its capabilities extend to real-time adaptations of treatment regimens. Take peritoneal dialysis as an example: conventional methods often depend on a rigid schedule and formulation of dialysate. Chain-of-thought prompting could dynamically adjust dialysate composition and dwell times based on real-time lab results. The system could ingest new lab data as they come in, adjust its recommendations in response to changes, and, thus, actively contribute to minimizing complications, such as peritonitis, while optimizing treatment effectiveness. 

### 6.3. Medication Optimization

The application of chain-of-thought prompting extends its utility to complex medication management scenarios. Particularly in cases like transplant recipients on immunosuppressants, medication management is an intricate dance of competing priorities: balancing efficacy with the potential for adverse reactions or drug–drug interactions. By maintaining a consistent and logical dialogue with the user, the system could suggest real-time medication adjustments based on new laboratory results, observed side-effects, or even reports from other healthcare providers. This dynamic adjustment has the potential to substantially optimize patient outcomes, reduce hospital readmissions, and improve overall quality of care.

### 6.4. Integration with Existing Clinical Systems and Electronic Health Records

AI models like LLMs are adept at handling complex datasets and numerous variables, outperforming traditional data analysis methods. Utilizing AI tools such as GPT streamlines database management significantly. Electronic Health Records (EHRs) have been a game-changer in healthcare, offering a comprehensive digital compilation of patient data [47]. In nephrology, for instance, EHRs encompass a range of clinical information pertinent to kidney health, including lab results, imaging studies, medication records, and progress notes. They bring numerous benefits, like instant access to patient data, aiding in clinical decisions, and bolstering research.

Integrating chain-of-thought LLMs into clinical systems and EHRs could revolutionize healthcare, leading to more informed and holistic medical care [48]. This integration involves a sophisticated combination of technology, data management, and system interoperability. Application Programming Interfaces (APIs) serve as the main technical pathway for merging LLMs with clinical systems. They facilitate the bidirectional flow of data between LLMs and EHRs, enabling efficient information exchange. Chain-of-thought LLMs, with their advanced natural language processing capabilities, can interpret clinical notes, patient histories, and diagnostic data, making sense of complex medical terminology and patient information. Notably, adherence to standards like HL7 (Health Level 7) and FHIR (Fast Health Interoperability Resources) is vital for ensuring accurate data interpretation across different EHR systems [49], alongside compliance with regulations such as HIPAA (Health Insurance Portability and Accountability Act) to protect data privacy and security [40]. In addition, scalability is also key to managing diverse data volumes and queries, and minimal latency is essential for real-time response generation in clinical environments. Chain-of-thought LLMs offer thorough, step-by-step analysis in clinical settings, assisting in differential diagnosis, treatment planning, and risk assessment. Customizing this process for specific clinical scenarios enhances both the relevance and accuracy. A feedback mechanism whereby clinicians review and refine the AI’s reasoning further improves model reliability.

Thus, the integration of LLMs, especially those utilizing chain-of-thought reasoning, holds immense promise for enhancing clinical decision making. These models can dissect complex medical situations, providing valuable support to healthcare professionals. Before widespread implementation, thorough testing and validation in real-world medical environments are crucial to ensure their accuracy and efficacy. Ensuring patient safety and privacy is a top priority, and LLM recommendations should always be interpreted by qualified professionals. Effective integration also hinges on educating healthcare providers on the effective use of these systems, understanding their strengths and limitations. Despite their advanced capabilities, LLMs are not a replacement for human judgment but a support tool to augment clinical expertise.

### 6.5. Continuous Learning and Model Updating

Nephrology is a rapid advancing field, characterized by remarkable technological innovations, major research achievements, and a transition towards holistic and patient-focused care. This vigorous progression demonstrates the dual aspects of challenges and possibilities in comprehending, treating, and managing kidney-related ailments. Keeping up with updates and performing regular maintenance of an LLM system in this rapidly evolving field is crucial, involving a multifaceted approach. 

Periodically retraining the LLM with the latest medical literature, research findings, and clinical guidelines in nephrology ensures that the model’s knowledge base remains current. In addition, integrating new and diverse data sources, such as recent nephrology journals, conference proceedings, and clinical trial reports, can provide the LLM with up-to-date information. Moreover, ensuring the LLM system is seamlessly integrated with EHRs allows for real-time updates on new treatments, patient outcomes, and research in nephrology. Implementing a system where clinicians can provide feedback on the LLM’s performance and suggestions can also help identify areas needing updates.

Furthermore, regular consultations with nephrology experts can guide the updating process, ensuring the LLM is aligned with current clinical practices and emerging trends. Involving the LLM in continuing medical education activities, like webinars and workshops, can also aid in keeping it abreast of new developments. Additionally, regular software updates are crucial to address security vulnerabilities, improve functionality, and enhance the AI’s processing capabilities. Ensuring the system architecture is scalable and adaptable allows for efficient incorporation of new features and capabilities as the field evolves. Continuous investment in research and development can lead to innovations in how the LLM is applied within nephrology. Engaging in collaborative research with academic institutions or other organizations can bring fresh perspectives and ideas for system improvement. Regular training for healthcare professionals using the LLM ensures they are up to date with its capabilities and can use it effectively. A robust support system helps address user issues and gathers insights for future updates.

Notably, keeping track of changes in healthcare regulations like HIPAA and data privacy laws ensures the LLM remains compliant. Regularly reviewing and updating the system to ensure ethical AI practices, particularly in terms of fairness, transparency, and nonbias.

Therefore, maintaining an LLM system in a dynamic field like nephrology requires an ongoing commitment to learning, collaboration, and technological adaptation. By incorporating these strategies, an LLM can remain a valuable and current tool in the rapidly advancing world of nephrology.

## 7. Ethical Consideration

In situations involving ethical complexities, such as end-of-life care or emergency resource distribution, chain-of-thought prompting can be fine-tuned to align with ethical standards [26]. This is essential to guarantee the safety, reliability, efficacy, and privacy of the technology. Compliance with healthcare regulations and standards such as HIPAA in the United States, GDPR (General Data Protection Regulation) in the European Union, and other similar regulations worldwide is a critical consideration when integrating LLMs like GPT into nephrology and other medical fields [50,51]. These regulations mandate strict confidentiality and security of patient health information. When using LLMs in nephrology, it is essential to ensure that patient data are anonymized or de-identified before being processed by the AI to prevent any privacy breaches. Under GDPR, and to some extent under HIPAA, there is an emphasis on obtaining explicit consent for data processing. Patients should be informed about how their data are used, including any AI involvement in their care. In addition, these regulations advocate for using the minimum amount of data necessary for the intended purpose. This principle should guide the integration of LLMs in clinical settings, ensuring that only relevant data are processed for specific medical inquiries. 

Both HIPAA and GDPR stress the importance of safeguarding data through encryption and strict access controls [52]. When LLMs access EHRs or other medical databases, it is crucial to employ robust encryption and limit access to authorized personnel only. Conducting regular security audits and assessments can help in identifying and mitigating potential vulnerabilities in the system, thus maintaining compliance with these regulations.

Maintaining detailed logs and audit trails of how patient data are accessed and used by the LLM is crucial for compliance. It helps in tracking and reporting any data breaches or unauthorized access. In the event of a data breach or noncompliance, regulations like HIPAA and GDPR require timely reporting to the relevant authorities and, in some cases, to the affected individuals. Under GDPR, and as a best practice, in general, conducting a Data Protection Impact Assessment (DPIA) for LLM integration can identify risks to patient data privacy and help in implementing appropriate safeguards. Keeping comprehensive documentation of data processing activities, including the purpose and legal basis for processing, is essential for compliance.

Beyond legal compliance, ethical considerations around fairness, nondiscrimination, and transparency in AI decision making are important, especially in sensitive areas like healthcare [48,50]. Notably, both healthcare regulations and AI models particularly the latter are continually evolving. Regularly updating the AI system and its operational protocols to align with the latest regulatory changes is essential.

The integration of LLMs in nephrology, while promising for enhancing patient care and medical research, requires careful adherence to healthcare regulations and standards. Balancing the innovative potential of AI with the stringent demands of data privacy, security, and ethical use is crucial. Continuous vigilance, regular updates to the system and practices, and a clear understanding of regulatory requirements are key to successfully leveraging AI in healthcare while maintaining compliance.

The intersection of AI systems with critical aspects of human life underscores the need for ethically guided decision making. By customizing chain-of-thought prompting to conform to ethical protocols, LLMs become more trustworthy and ethically sound in their responses, addressing a crucial aspect of AI deployment in sensitive and impactful domains. 

## 8. Conclusions

Chain-of-thought prompting has shown great promise as an innovative approach to personalized diagnosis and treatment in nephrology. By enabling a more nuanced, individualized approach, this mechanism has the capacity to drive substantial improvements in patient outcomes. The real-time adaptation of treatment plans and medication regimes sets the stage for a revolutionary change in managing renal conditions.

This review uniquely applies chain-of-thought prompting in LLMs to nephrology, a field that presents distinct challenges. This targeted application is relatively unexplored in the existing literature, positioning our work at the forefront of AI utilization in kidney disease management. The comparative analysis is instrumental in understanding the effectiveness of different AI approaches in addressing complex medical scenarios specific to kidney diseases. The review also delves into the technical intricacies of LLMs, including algorithmic implementation and integration with clinical systems. This detailed exploration contributes to a deeper understanding of how AI can be effectively and safely integrated into healthcare practices, particularly in nephrology. Moreover, by rigorously analyzing the ethical implications of using LLMs in nephrology, we address a critical gap in the current discourse. This aspect is particularly important given the sensitive nature of medical data and the ethical responsibility in healthcare decision making. It is important to note that this review goes beyond the current applications and explores the future potential of chain-of-thought prompting in nephrology. The significance of this work lies in its forward-looking perspective to enhance diagnosis, treatment planning, and patient care in kidney disease, thereby contributing to improved health outcomes.

However, there are challenges to using this method in nephrology, like ensuring data safety, ethical concerns about AI in healthcare, and the need for large clinical trials to test it. It is also important to have clear rules about using AI in medical decisions. Despite these issues, the future looks bright for chain-of-thought prompting, with many opportunities for innovation. For example, combining it with Internet of Things (IoT) medical devices, like advanced dialysis machines, could allow for instant treatment changes based on real-time data. Also, merging this method with genomics could lead to more precise treatments for kidney diseases. Overall, with careful research and use, LLMs with chain-of-thought prompting could greatly change nephrology for the better.

## Data Availability

Availability Statements are available in the original publication, reports, and preprints that were cited in the reference citation.

## Supplementary Materials

The following supporting information can be downloaded at: https://www.mdpi.com/article/10.3390/medicina60010148/s1, Table S1: Responses to Direct Prompt in Google Bard AI and AI21 Studio Models; Table S2: Google Bard AI’s Responses to Chain-of-thought Prompting for Example 1; Table S3: AI21’s Responses to Chain-of-thought Prompting for Example 1; Table S4: Google Bard AI’s Responses to Chain-of-thought Prompting for Example 2; Table S5: AI21’s Responses to Chain-of-thought Prompting for Example 2.
