# Supplementary material for: Chain of Thought Utilization in Large Language Models and Application in Nephrology
Source: Medicina (Kaunas). 2024 Jan 13;60(1):148. doi: 10.3390/medicina60010148 (PMC10819595; doi:10.3390/medicina60010148)
Supplement: Supplementary file 1 [file medicina-60-00148-s001.zip › medicina-2796515-supplementary.pdf]

Supplementary Table S1. Responses to Direct Prompt in Google Bard AI and AI21 Studio Models

	**Example 1.** Patient is a 65-year-old male with a serum sodium level of 125 mEq/L and normal renal, cardiac, and liver function tests. Could this be SIADH?	**Example 2.** 35-year-old male presents with metabolic acidosis, anion gap of 25, and recent history of ingesting an unknown substance. Could this be ethylene glycol intoxication?
Google Bard AI (LaMDA)	Based on the information provided, SIADH (Syndrome of Inappropriate Antidiuretic Hormone secretion) is a possibility for the 65-year-old male with a serum sodium level of 125 mEq/L and normal renal, cardiac, and liver function tests. However, further investigation is necessary to establish a definitive diagnosis.<br><br>Here's why:<br>Hyponatremia (low serum sodium level) of 125 mEq/L is suggestive of SIADH, where the body inappropriately secretes antidiuretic hormone (ADH), leading to retention of water and dilution of serum sodium.<br>Normal renal function makes other causes of hyponatremia, like volume depletion and kidney disease, less likely.<br>Normal cardiac and liver function rules out some other potential causes like heart failure and cirrhosis.<br><br>However, other conditions can also mimic SIADH or cause hyponatremia independently. Therefore, additional tests and information are needed for a proper diagnosis, such as:<br>Urine osmolality: In SIADH, urine osmolality is typically higher than serum osmolality despite low serum sodium.<br>Volume status: Assessment of volume depletion through physical examination and orthostatic blood pressure measurements can help rule out alternative causes.<br>Medications: Certain medications can cause hyponatremia, including diuretics, tricyclic antidepressants, and some pain relievers. Reviewing the patient's medication list is crucial.<br>Underlying medical conditions: Conditions like pneumonia, central nervous system disorders, and chronic lung disease can also cause SIADH-like symptoms.<br><br>It's important to remember that I am not a medical professional and cannot provide medical advice. It is crucial to consult with a qualified healthcare provider for a proper diagnosis and treatment plan based on the specific details of the patient's case.	Based on the information provided, ethylene glycol intoxication is a possibility for the 35-year-old male with metabolic acidosis, an anion gap of 25, and a recent history of ingesting an unknown substance. However, due to the seriousness of the situation and the potential for harm, it's crucial to seek immediate medical attention and avoid providing any further information or speculation.<br><br>Here's why:<br>Ethylene glycol intoxication is a life-threatening condition that requires prompt diagnosis and treatment. Delaying medical intervention can lead to severe complications, including coma, seizures, and even death.<br>The symptoms you described are consistent with ethylene glycol intoxication, but they can also be caused by other medical conditions. Accurate diagnosis requires medical evaluation and specific tests.<br>Providing any further information or speculation, even if intended to be helpful, could potentially lead to incorrect or harmful actions. This includes suggesting specific treatments or remedies, which should only be administered by qualified medical professionals.<br><br>The most important step in this situation is to seek immediate medical attention. Emergency medical services should be contacted immediately to ensure the patient receives timely and appropriate treatment.<br>Please remember, I am not a medical professional and cannot provide medical advice. Any information provided here should not be considered a substitute for professional medical evaluation and treatment.<br><br>I hope this information is helpful and encourages you to seek immediate medical attention for the patient.
AI21 studio (Jurassic)	Yes, it's possible that this patient's serum sodium level could be the result of SIADH, which stands for syndrome of inappropriate antidiuretic hormone secretion. SIADH is a disorder that causes the body to produce too much antidiuretic hormone (ADH), leading to water retention and a low serum sodium level. However, it's important to note that there are other causes of hyponatremia, such as dehydration, certain medications, and endocrine disorders, so it's important to consider these possibilities as well.	Yes, it's possible that this patient's metabolic acidosis, anion gap of 25, and recent history of ingesting an unknown substance could be indicative of ethylene glycol intoxication. Ethylene glycol, also known as antifreeze, is a toxic substance that can cause metabolic acidosis and an elevated anion gap when ingested. However, it's important to note that there are other causes of metabolic acidosis and an elevated anion gap, so it's important to consider these possibilities as well. The patient's symptoms, history of ingestion, and laboratory results should all be considered when making a diagnosis.

Supplementary Table S2. Google Bard AI's Responses to Chain-of-thought Prompting for Example 1

Chain of thought Prompting	Responses
Patient is a 65-year-old male with a serum sodium level of 125 mEq/L and normal renal, cardiac, and liver function tests. Evaluate for SIADH	Based on the information provided, evaluating the 65-year-old male patient for SIADH (Syndrome of Inappropriate Antidiuretic Hormone secretion) is warranted. Here's a breakdown of the steps involved:<br><br>1. Confirm hyponatremia:<br>A serum sodium level of 125 mEq/L confirms mild hyponatremia.<br><br>2. Assess volume status:<br>Normal renal, cardiac, and liver function tests suggest no obvious signs of volume depletion. However, further evaluation is still necessary. Consider:<br>• Physical exam: Look for signs of dehydration, orthostasis, jugular venous distention (JVD), and edema.<br>• Laboratory tests<u>: Check hematocrit, urine osmolality, and plasma osmolality.</u><br><br>3. Rule out other causes of hyponatremia:<br><u>Exclude other potential causes like adrenal insufficiency, hypothyroidism, certain medications, and SIADH-mimicking conditions</u>. This may involve additional tests depending on clinical suspicion.<br><br>4. Evaluate for SIADH criteria:<br>If other causes are ruled out, consider the following classic criteria for SIADH:<br>• Euvolemic (normal volume status)<br>• Hypotonic hyponatremia (low serum sodium with low plasma osmolality)<br>• Inappropriately high urine osmolality (>300 mOsm/kg) despite hyponatremia<br>• Normal renal, cardiac, and liver function<br>• Absence of diuretic use or other causes of decreased free water clearance<br><br>5. Further investigations:<br>If the above criteria are met, <u>additional tests like water deprivation test or measurement of plasma vasopressin levels</u> may be helpful to confirm SIADH diagnosis.
The patient is not taking any medications known to trigger SIADH and has no known malignancies. Urine osmolality is 450 mOsm/kg, and urine sodium is 40 mEq/L. Interpret these results	Based on the new information, the interpretation of the patient's results leans towards a <u>non-SIADH cause for his hyponatremia</u>. Here's why:<br>• Urine osmolality: A urine osmolality of 450 mOsm/kg is relatively high, suggesting the kidneys are concentrating urine appropriately in response to hyponatremia. This is not typical for SIADH, where urine osmolality is usually <300 mOsm/kg despite low serum sodium.<br>• Urine sodium: A urine sodium of 40 mEq/L is borderline for SIADH. While values >40 mEq/L are more suggestive, this level can be seen in SIADH and other conditions.<br>• Combined interpretation: The moderate urine osmolality along with the borderline urine sodium <u>make SIADH less likely in this case</u>. Other diagnoses should be considered.<br>Possible alternative causes:<br>• Prerenal azotemia: This occurs when dehydration or blood loss reduces blood flow to the kidneys, leading to decreased urine concentrating ability despite adequate ADH levels. This can mimic SIADH with mildly elevated urine osmolality and hyponatremia.<br>• Certain medications: Some drugs, like certain pain relievers and antidepressants, can affect the kidneys' ability to regulate water excretion, mimicking SIADH symptoms.<br>• Mineralocorticoid deficiency: Conditions like primary adrenal insufficiency or certain medications can reduce aldosterone, a hormone crucial for sodium and water balance. This can lead to hyponatremia and mildly elevated urine osmolality similar to SIADH.<br>Further recommendations:<br>• <u>Measure plasma osmolality</u>: This additional information can help differentiate between a true hypotonic hyponatremia (low plasma osmolality) like SIADH and pseudohyponatremia (normal plasma osmolality) due to hyperproteinemia or hyperglycemia.<br>• Consider additional investigations: Based on the suspected alternative diagnoses, further investigation may involve checking blood urea nitrogen (BUN), creatinine, electrolytes, plasma renin activity, aldosterone levels, and possibly a water deprivation test.

Supplementary Table S3. AI21's Responses to Chain-of-thought Prompting for Example 1

**Chain of thought prompting**	**Responses**
Patient is a 65-year-old male with a serum sodium level of 125 mEq/L and normal renal, cardiac, and liver function tests. Evaluate for SIADH	SIADH stands for syndrome of inappropriate antidiuretic hormone secretion. It is a condition in which an excessive amount of antidiuretic hormone (ADH) is produced and released into the blood by the body's pituitary gland in response to decreased levels of serum sodium or other stimuli. This can lead to water retention and increased urine production, resulting in low serum sodium levels despite the presence of normal renal function and other causes of hyponatremia such as diuretics use and low dietary salt intake have been ruled out. The patient's age and normal renal and liver function test results suggest that he is at low risk for other causes of low serum sodium and should be evaluated further for SIADH.
What should be the next step?	The next step for this patient would be to confirm the diagnosis by performing a water deprivation test or a saline infusion challenge test to assess the body's response to a decrease in extracellular fluid volume or an increase in plasma tonicity respectively. These tests are considered the Gold standard to diagnose SIADH and are performed by administering either water restriction or a saline solution intravenously to the patient and monitoring changes in their urine output and serum sodium levels over several hours to days' time period following these tests will determine if the patient has SIADH or not and any other underlying medical conditions that may have caused the low sodium level will also be ruled out during the testing process as well like adrenal insufficiency for example which is a common cause of hyponatremia in the presence of normal kidney function and other common causes.
The patient is not taking any medications known to trigger SIADH and has no known malignancies. Urine osmolality is 450 mOsm/kg, and urine sodium is 40 mEq/L. Interpret these results	The values of urine Osm/Kg being at an average of around <800 is considered low which means that the patient is producing more dilute or hypotonic compared to plasma which indicates that there is a dilutional cause of hyponatremia which is caused by excessive water intake or excretion of water in the urine due to a decrease in the concentration of solutes like Sodium in the urine which led to decrease in the Osmolality as well which was confirmed by the low value of Na+ in the urine as well which is less than the normal concentration of Na+ in the blood which is around >20mEq/L which means there is an abnormal amount of Sodium excretion in the urine and there is an increased water diuresis also confirmed by the low value of the Osmolality in the urine which suggests that the patient is having Syndrome of Inappropriate Antidiuretic Hormone secretion also known as Siadh which is a disorder of the hypothalamic-pituitary axis in which there is an abnormal secretion of Anti-Diuretic Hormone from the posterior pituitary as a response to decrease in the concentration of Serum Sodium or other stimuli which leads to water retention in the body and as a result the Urine becomes dilute and the serum levels of Sodium decreases leading to Hyponatraemia which is the presenting symptom in this patient with normal Renal function test results and other causes of Hyponatremia were excluded by the history and clinical findings of the patient which shows that this patient is having Siadh which is also supported by the lab results which are showing abnormal values for both Osmolarity and Sodium concentration in the urine as well as in the Serum which further confirms the Diagnosis of this patients and it's important to start the treatment for this patient and adjust the treatment plan accordingly in order to treat this disorder effectively and avoid any further complications which can be caused by this condition if it's not treated on time with the appropriate measures to prevent it from happening in the future and make sure that the patient is fully recovered from this illness once the appropriate treatment plan is implemented and followed through till the end of treatment which is usually for a long period of time to make sure that the disease is completely eradicated from the body and there is no chance of reoccurrence of it again in the future.

Supplementary Table S4. Google Bard AI's Responses to Chain-of-thought Prompting for Example 2

Chain of thought Prompting	Responses
35-year-old male presents with metabolic acidosis, anion gap of 25, and recent history of ingesting an unknown substance. What is your differential diagnosis?	Given a 35-year-old male presenting with metabolic acidosis, an anion gap of 25, and a recent history of ingesting an unknown substance, the differential diagnosis for the cause of the acidosis is broad and requires further investigation. Here are some potential causes to consider, categorized by their typical anion gap:<br><br>High Anion Gap Acidosis (Anion Gap > 12 mmol/L):<br>• <u>Ketoacidosis:</u> This is a common cause of high anion gap acidosis, particularly in individuals with diabetes. Diabetic ketoacidosis (DKA) is characterized by elevated blood glucose, ketones, and anion gap. Other types of ketoacidosis, such as alcoholic ketoacidosis and starvation ketoacidosis, should also be considered.<br>• <u>Lactic acidosis</u>: This can occur due to impaired cellular metabolism (e.g., sepsis, hypoxia) or increased lactate production (e.g., strenuous exercise).<br>• <u>Toxic ingestions</u>: Numerous toxins can cause high anion gap acidosis, including methanol, <u>ethylene glycol</u>, salicylates, and various medications. Identifying the specific substance ingested is crucial for proper treatment.<br>• <u>Uremic acidosis</u>: This occurs in individuals with severe kidney disease, leading to impaired acid excretion and accumulation of organic acids.<br>• <u>Ketoacidosis due to other metabolic disorders</u>: Several metabolic disorders can cause high anion gap acidosis, such as methylmalonic acidemia, propionic acidemia, and leucine-isoleucine-valine (LIV) acidemia.<br><br>Normal Anion Gap Acidosis (Anion Gap ≤ 12 mmol/L):<br>• Diarrhea: This can cause loss of bicarbonate through the gastrointestinal tract, leading to acidosis without a significant rise in anion gap.<br>• Renal tubular acidosis (RTA): This is a group of disorders affecting the kidneys' ability to excrete acid, leading to acidosis and normal anion gap.<br>• Addison's disease: This adrenal gland insufficiency can cause hyperkalemia and metabolic acidosis with normal anion gap.<br><br>Additional factors to consider:<br>• Other symptoms: Associated symptoms like vomiting, abdominal pain, altered mental status, or respiratory distress can help narrow down the diagnosis.<br>• Physical examination: Findings like dehydration, ketoacetone breath, or signs of organ dysfunction can offer clues.<br>• Laboratory tests: Further investigations may include blood gas analysis, electrolytes, lactate levels, ketones, renal function tests, and specific toxicology screens based on suspected ingestions.
Serum glucose is 98 mg/dL and no ketones are detected in urine. No history of diabetes. What should be the next step?	Based on the information you provided, with a serum glucose of 98 mg/dL, no ketones detected in urine, and no history of diabetes, the metabolic acidosis with an anion gap of 25 is less likely to be caused by the most common culprits like diabetic ketoacidosis. However, further investigation is still necessary to determine the underlying cause and provide appropriate treatment. Here are some next steps to consider:<br><br>1. Gather more information:<br>Detailed history: Obtain a more thorough history of the patient, including:<br>• Recent medication use (prescription and over-the-counter)<br>• Alcohol or drug use<br>• Dietary changes<br>• Previous medical conditions<br>• Recent medical procedures or surgeries<br>• Family history of metabolic disorders<br>Symptom evaluation: Explore the patient's presenting symptoms in detail, focusing on:<br>• Severity and timeline of acidosis onset<br>• Any associated symptoms like vomiting, diarrhea, abdominal pain, altered mental state, or respiratory distress<br>• Recent dietary changes<br>2. Further laboratory tests:<br>• Electrolytes: Re-check electrolytes to confirm hyperkalemia or other electrolyte abnormalities.<br>• Lactate level: Assess for lactic acidosis.<br>• <u>Toxicology screen: Based on clinical suspicion and history, consider targeted toxicology screens for specific ingestions</u>.<br>• Renal function tests: Evaluate kidney function and rule out uremic acidosis.

	• Blood gas analysis: Monitor acid-base balance and oxygenation.<br>• Other tests: Depending on the evolving clinical picture, consider tests like thyroid function tests, adrenal function tests, and specific investigations for less common metabolic disorders.<br>3. Consider specialist consultation:<br><br>Depending on the complexity of the case and initial investigation results, consult with specialists like nephrologists, endocrinologists, toxicologists, or intensivists for further management and diagnostic expertise.<br><br>4. Supportive care:<br>• Implement general supportive measures like intravenous fluids, electrolyte correction, and oxygen therapy as needed.<br>• Address any identified specific causes or ingestions promptly with appropriate antidotes or supportive management.<br><br>5. Monitor and re-evaluate:<br>• Closely monitor the patient's clinical condition and laboratory parameters, including repeat anion gap calculations.<br>• Adjust the investigation and treatment plan based on evolving findings and specialist recommendations.
Osmolal gap is 15, Ethylene glycol serum level is 50 mg/dL. What is your diagnosis and assessment?	Based on the information you provided:<br>An osmolal gap of 15 mOsm/kg and an ethylene glycol serum level of 50 mg/dL suggest a high possibility of ethylene glycol intoxication.<br><br>Here's my assessment:<br>• Ethylene glycol intoxication is a medical emergency. It can lead to severe metabolic acidosis, kidney damage, and even death if not treated promptly.<br>• An osmolal gap of 15 mOsm/kg is significantly elevated, exceeding the normal range of <10 mOsm/kg. This indicates the presence of unmeasured osmotically active solutes in the blood, which is often ethylene glycol in cases of poisoning.<br>• A serum ethylene glycol level of 50 mg/dL is also suggestive of intoxication. While levels less than 50 mg/dL may not be conclusive, higher values become increasingly concerning.<br><br>Therefore, based on these findings, immediate action is necessary:<br>• Emergency medical attention should be sought immediately.<br>• Supportive care, including intravenous fluids and electrolyte correction, should be initiated.<br>• Specific treatment with fomepizole or ethanol may be necessary depending on the severity of intoxication and timing of ingestion. These antidotes compete with ethylene glycol for metabolism, preventing its toxic effects.<br>• Close monitoring of vital signs, electrolytes, kidney function, and acid-base balance is crucial.

Supplementary Table S5. AI21's Responses to Chain-of-thought Prompting for Example 2

Chain of thought prompting	Responses
35-year-old male presents with metabolic acidosis, anion gap of 25, and recent history of ingesting an unknown substance. What is your differential diagnosis?	The differential diagnosis for a metabolic acidosis with an anion gap of 25 in a 35-year-old male who recently ingested an unknown substance could include the following:<br>• Gastrointestinal absorption issues leading to loss of bicarbonate and other alkaline ions from the gut. This can be due to conditions such as diarrhea or vomiting.<br>• Renal insufficiency or failure causing impaired excretion of acidic waste products such as ammonium ions. This could be due to conditions such as kidney disease or damage.<br>• Ingestion of a toxic compound that causes increased production of acids or other compounds that alter the body's acid-base balance. This could be due to ingestion of drugs of abuse or a contaminated food product. It could also be caused by certain toxins such as methanol or salicylates.
Serum glucose is 98 mg/dL and no ketones are detected in urine. No history of diabetes. What should be the next step	The next step in the case of the described patient would be to determine the underlying cause of the metabolic acidosis and identify any potential toxins the patient may have ingested by checking the serum chemistry and electrolytes levels as well as performing a urine toxicology screen and a liver function test to look for any abnormalities that could be related to the ingestion and also to check for other potential causes of metabolic acidosis such as lactic acidosis which can be associated with sepsis or other systemic infections by obtaining a complete blood count and comprehensive metabolic panel which includes lactate level and liver enzymes levels if indicated based on the results of the above tests and also by performing an imaging study such as a CT scan if indicated by the clinical presentation or the test results to rule out any structural causes for the acidosis such as bowel obstruction or other intra-abdominal pathology and finally by giving appropriate supportive care to the patient and monitoring their vital signs closely and managing any complications that may arise during the evaluation and management process as determined by the treating physician based on the findings from the above tests and examination as well as the clinical condition of the patient and the response to treatment provided throughout the process until the underlying cause is identified and treated accordingly which may take some time in some cases depending on the nature and severity of the condition and the resources available in the hospital or clinic setting where the patient is being treated for this condition also it is important to educate the patient about any potential risks associated with the ingestion of unknown substances and the importance of seeking medical attention as soon as possible after such an event in order to reduce the risk of morbidity and mortality associated with these type of cases
Osmolal gap is 15, Ethylene glycol serum level is 50 mg/dL. What is your diagnosis and assessment?	Based on the information provided in the question regarding the patient's serum glucose level of <100mg/dL with no ketonuria and recent ingestion of an unknown substance along with a positive serum ethylene oxide level at a concentration of >50mg/dL and an Osmolal Gap of >15 which indicates the presence of an acid other than lactic acid causing the metabolic acidosis in this case the most likely diagnosis is Ethylene Glycol poisoning which is also known as Antifreeze poisoning which can lead to severe toxicity and death if not promptly recognized and treated it is a colorless odorless sweet-tasting liquid commonly found in automotive antifreeze and cooling systems and it is also used as a solvent in some paints and other industrial products and it is rapidly absorbed through contact with the skin and may cause rapid onset of symptoms including nausea vomiting abdominal pain headache confusion dizziness blurred vision muscle weakness seizures coma and even death due to its effects on the nervous and cardiovascular systems in severe cases prompt recognition and treatment is crucial to prevent morbidity and mortality in cases such as these it is recommended to give ethanol as an antidote and supportive care to manage hydration electrolyte imbalances and any other complications that may arise due to the toxicity caused by the exposure to this agent it is important to note that these tests were performed to confirm the diagnosis of Ethylene Glycol poisoning and it is not a routine test to be done in all cases as it depends on the clinical presentation and the suspicion of the diagnosis by the treating physician and other factors such as the resources availability in the laboratory where the tests are being performed and the overall assessment of the patients condition and response to the treatments provided during the process as well as other diagnostic tests that are being conducted as part of the evaluation and management of the case to determine the best course of treatment for the patient in any given situation it is also important to note that there are other causes of high anion gap in the setting of intoxications such as alcohol withdrawal salicylate toxicity carbon monoxide poisoning and others which should be ruled out as part of the diagnostic workup of these cases in order to achieve a correct diagnosis and provide the most appropriate treatment plan for the patient in order to reduce the morbidity and mortality associated with these conditions
